# Efficiency and Safety of Chinese Herbal Medicine in the Treatment of Prediabetes: A Systemic Review and Meta-Analysis of Randomized Controlled Trials

**DOI:** 10.1155/2020/3628036

**Published:** 2020-10-14

**Authors:** Yanan Song, Haiyan Wang, Lingling Qin, Mei Li, Saiyue Gao, Lili Wu, Tonghua Liu

**Affiliations:** ^1^The School of Life Sciences, Beijing University of Chinese Medicine, Beijing 100029, China; ^2^Beijing International Technology Cooperation Base for Prevention and Treatment of Diabetes Mellitus with Chinese Medicine, Beijing 100029, China; ^3^Health-Cultivation Laboratory of the Ministry of Education, Beijing University of Chinese Medicine, Beijing 100029, China; ^4^Physical Examination Laboratory, Linyi Lanshan District Center for Disease Control and Prevention, Linyi, Shandong 276000, China

## Abstract

**Objective:**

The aim of this study was to review existing evidence on the efficiency and safety of Chinese herbal medicine for the treatment of prediabetes.

**Methods:**

Randomized controlled trials (RCTs) of Chinese herbal medicine (CHM) to treat prediabetes were searched in the following databases from their inception date onwards until 2 May 2020: MEDLINE, Cochrane, EMBASE, Web of Science, EBSCO, CINAHL, CNKI, VIP database, CBM, and Wanfang database. Quality assessment of included trials was accessed according to the guidance in Cochrane. Researchers independently assessed the validity of included trials and extracted outcome data for synthesis. RevMan 5.3 was used for the meta-analysis.

**Results:**

Twenty-two RCTs including 3923 participants were included in the study. Our findings upon the 22 RCTs showed CHM is effective in the treatment of prediabetes, which can statistically reduce the incidence of diabetes (RR = 0.48; 95% CI = (0.41, 0.57); *P* < 0.001), increase the incidence of normalization of prediabetes (RR = 1.76; 95% CI = (1.57, 1.96); *P* < 0.001), and lower FPG (MD = −0.38; 95% CI = (−0.60, −0.16); *P* < 0.001), 2hPG (MD = −1.13; 95% CI = (−1.60, −0.67); *P* < 0.001), TG (MD = −0.23; 95% CI = (−0.33, −0.13); *P* < 0.001), TC (MD = −0.34; 95% CI = (−0.52, −0.16); *P* < 0.001), and BMI (MD = −0.48; 95% CI = (−0.78, −0.18); *P* < 0.001) after treatment, and there was no difference of HbA1c (*P* > 0.05).

**Conclusion:**

CHM is effective for the treatment of prediabetes. CHM can statistically reduce the incidence of diabetes, increase the incidence of normalization of prediabetes, and lower the FPG, 2hPG, TG, TC, and BMI levels, but with no significant difference in HbA1c. In addition, CHM was relatively safe in clinical practice. More high-quality RCTs should be conducted to strengthen the finding.

## 1. Introduction

Prediabetes, also named impaired glucose regulation (IGR), is an abnormal glucose metabolism period between normal blood glucose and diabetes, including mild impaired fasting glucose (IFG) and/or impaired glucose tolerance (IGT) [1–4]. The latest IDF diabetes map released in 2019 indicates that, among 463 million diabetes patients, 231.9 million (50.1%) are undiagnosed, and compared with 2017, IGT population increased by 22 million [5]. The risk of diabetes is greatly increased in subjects with prediabetes. Prediabetes period could exist for many years with no obvious symptoms of diabetes, and by the time type 2 diabetes is diagnosed, half of the people may suffer from diabetes-related tissue damage. Furthermore, most prediabetes patients have obvious insulin resistance [6]. With the aggravation of insulin resistance and the failure of islet *β-*cell function, blood sugar is gradually difficult to control, leading to a high risk of microvascular and macrovascular injuries, which are similar to chronic complications of diabetes [7]. To be more specific, nearly 10% of people with IFG and IGT progress to diabetes every year [8], and the recent research suggests that 93% of subjects with prediabetes may develop diabetes within 20 years without active intervention [9]. However, with at least six years of lifestyle interventions, the incidence of diabetes could drop by 43% [10]. Thus, preventing the transition of prediabetes to T2DM represents an important approach in combating the T2DM pandemic.

Pharmacological interventions have been used to treat prediabetes, such as metformin, acarbose, and troglitazone [11, 12]. However, modern medicine, which focuses on regulating blood sugar, has certain limitations in the treatment of diabetes and its complications, and some adverse reactions may occur after long-term administration [13]. For example, the use of thiazolidinedione drugs is often accompanied by fractures and side effects of cardiovascular and cerebrovascular diseases [13, 14]; biguanides have gastrointestinal side effects [15], and patients who are intolerant to biguanides are at risk of using insulin sensitizers.

Chinese herbal medicine (CHM) has been used to treat IGT, IFG, and diabetes for a long time [16]. Several clinical trials have proved that CHM has advantages in blood sugar control, and its curative effect has been widely recognized. Compared with conventional hypoglycemic drugs, most CHMs have lower cost, fewer adverse reactions, and are more effective for certain specific complications [17, 18]. Many studies show that CHM contains many active ingredients, which can provide many therapeutic effects for many targets, such as enhancing insulin sensitivity [19], stimulating insulin secretion [20], controlling inflammation [21], and regulating glucose absorption [22]. For example, berberine is the main component of *Coptis*, and its ability to improve glycolipid metabolism has been confirmed in clinical trials [23]. In addition, berberine can also inhibit islet cell apoptosis [24], reduce oxidative stress [25], regulate intestinal microflora [26], etc.

Being a widely practiced and long-time-used medical method, CHM has been proved to be effective in prediabetes treatment; however, there was no sufficient evidence-based medicine (EBM) support of that for clinicians and specialists. Consequently, the aim of this study was to perform a systematic review and meta-analysis of RCTs for patients with prediabetes and to address the questions regarding whether CHM is effective and safe as an adjunctive therapy for managing prediabetes.

## 2. Materials and Methods

This protocol was registered with the International Platform of Registered Systematic Review and Meta-Analysis Protocols (INPLASY). The registration number was INPLASY202050015. This article was written following the Preferred Reporting Items for Systematic Reviews and Meta-Analyses (PRISMA [27]) reporting guidelines.

### 2.1. Search Strategy

The following electronic databases were searched to identify eligible trials published from their inception date onwards until 2 May 2020. The English electronic databases included MEDLINE, the Cochrane Central Register of Controlled Trials, EMBASE, Web of Science, EBSCO, and CINAHL database; the Chinese electronic databases were the Chinese National Knowledge Infrastructure Database (CNKI), the Chinese Biomedical Database (CBM), Wanfang database, and VIP Chinese Science and Technique Journals Database. In addition, trial registers were also searched: World Health Organization International Clinical Trials Registry (http://www.who.int/ictrp/en/) and US National Institutes of Health Ongoing Trials Register (http://www.ClinicalTrials.gov). No language restriction was used. We further scanned the references of all included studies and relevant reviews to identify any trials that met our inclusion criteria. The retrieved articles were imported into EndNote X9 for document management and analysis.

Details of the search strategy are as follows:

(i) Search strategy (MEDLINE)
    #1 “Prediabetic State”[mh]
    #2 (Prediabetic State∗[tiab]) OR (State, Prediabetic[tiab]) OR (States, Prediabete∗[tiab]) OR (prediabete∗[tiab]) OR (pre-diabete∗[tiab]) OR (impaired glucose regulation[tiab]) OR (impaired glucose tolerance[tiab]) OR (impaired fasting glucose[tiab]) OR (reduced glucose regulation[tiab]) OR (reduced glucose tolerance[tiab]) OR (reduced fasting glucose[tiab]) OR (insulin resistance[tiab]) OR (impaired insulin[tiab]) OR (reduced insulin[tiab]) OR (Diabete∗ prevention[tiab])
    #3 #1 OR #2
    #4 (“Medicine, Chinese Traditional”[mh]) OR (“Drugs, Chinese Herbal”[mh]) OR (“Medicine, East Asian Traditional”[mh]) OR (“Materia Medica”[mh]
    #5 (Traditional Chinese Medicine[tiab]) OR (Traditional Medicine, Chinese[tiab]) OR (Chinese Traditional Medicine[tiab]) OR (Chinese Medicine, Traditional[tiab]) OR (Zhong Yi[tiab]) OR (Chinese Drug∗, Plant[tiab]) OR (Chinese Herbal Drug∗[tiab]) OR (Herbal Drug∗, Chinese[tiab]) OR (Plant Extract∗, Chinese[tiab]) OR (Chinese Plant Extract∗[tiab]) OR (Extract∗, Chinese Plant[tiab]) OR (Oriental Medicine, Traditional[tiab]) OR (Medicine, Traditional Oriental[tiab]) OR (Traditional Oriental Medicine[tiab]) OR (Traditional Far Eastern Medicine[tiab]) OR (Oriental Traditional Medicine[tiab]) OR (Oriental Medicine[tiab]) OR (Far East Medicine[tiab]) OR (Plants, Medicinal[tiab]) OR (Medicinal Plant∗[tiab]) OR (Pharmaceutical Plant∗[tiab]) OR (Medicinal Herb∗[tiab]) OR (Herb∗, Medicinal[tiab]) OR (Medica, Materia[tiab]) OR (Materia Medica[tiab])
    #6 #4 OR #5
    #7 (randomized controlled trial[pt] OR controlled clinical trial[pt] OR randomized[tiab] OR placebo[tiab] OR clinical trials as topic[mesh:noexp] OR randomly[tiab] OR trial[ti] NOT (animals[mh] NOT humans [mh]))
    #8 #3 AND #6 AND #7

### 2.2. Study Selection

#### 2.2.1. Types of Participants

We included participants of prediabetes patients who were clearly diagnosed by internationally recognized criteria. No sex or age limitation. The diagnosis criteria include WHO 1999, ADA 1999, and ADA 2008.

#### 2.2.2. Types of Intervention and Comparison

The CHM interventions included a Chinese proprietary medicine, or a compound of several herbs irrespective of preparation (e.g., tablet, decoction, oral liquid, pill, capsule, and powder), and the single herbs (including extracts from a single herb). We only include the oral delivery; we excluded intramuscular or intravenous. We did not restrict the dosage of herbs. We included trials if the treatment was given for a minimum of four weeks.

The control interventions were ① lifestyle management (LM; e.g., diet, education, and exercise); ② placebo; ③ no treatment; ④ conventional Western medicine (biguanides such as metformin); cointerventions were allowed as long as all arms of the randomized trial received the same cointervention. Only interventions performed for a minimum duration of four weeks were included.

#### 2.2.3. Types of Outcome Measures

The primary outcome includes ① incidence of T2DM, as diagnosed with at the time of the diagnosis prevailing diagnostic criteria; ② incidence of the normalization of blood glucose (the number of participants who returned to a normal blood glucose range by the end of the trial); ③ adverse events (AEs); ④ fasting plasma glucose (FPG); ⑤ 2-hour postprandial blood glucose (2hPG); ⑥ glycosylated hemoglobin levels A1c (HbA1c). The secondary outcome includes ⑦ triglycerides (TG); ⑧ total cholesterol (TC); ⑨ reduction in body mass index (BMI).

#### 2.2.4. Types of Study Designs to Be Included

We include randomize controlled trials (RCTs) irrespective of blinding or language. We excluded quasi-randomized trials.

### 2.3. Eligibility Criteria

All trials met the following eligibility criteria: ① the study was a randomized controlled trial (RCT); ② the study examined prediabetic participants who received oral CHM as intervention; ③ the study included participants irrespective of gender, age, or ethnicity, and prediabetes was diagnosed by clearly defined or internationally recognized criteria. For repeated studies, we contacted the correspondent to clarify the uncertainty. If the author cannot be contacted, the first published original document is considered. The exclusion criteria were ① comparative studies on different Chinese medicine therapies (e.g., CHM vs. acupuncture or moxibustion); ② abstracts or comments from conference papers; ③ studies that were nonrandomized controlled trials and quasi-randomized controlled trials.

### 2.4. Data Extraction and Management

Two review authors independently extracted data including details of the study population, intervention, and outcomes using a predesigned data extraction form. The following data were extracted: general trial characteristics (title, authors, and year); baseline of the patient and disease data (gender, age, and sample size); interventions (component and dose CHM and details of control interventions); and outcomes (outcome measures and adverse events). We resolved differences in data extraction by consensus or a third party. One author entered data into Cochrane software Review Manager 5.3 (RevMan 5.3) [28], and another checked the data to reduce the possibility of data entry errors.

### 2.5. Risk of Bias Analysis

Two authors independently assessed the “risk of bias” according to the guidance in the Cochrane Handbook for Systematic Reviews of Interventions [29]. This involved the following domains: random sequence generation, allocation concealment, blinding of participants, personnel and outcome assessors, incomplete outcome data, selective outcome reporting, and other sources of bias. We completed a “risk of bias” table according to Cochrane guidelines. We made judgement on each of these criteria relating to the risk of bias: low, high, or unclear (indicating unclear or unknown risk of bias). Discrepancies in this interpretation were resolved by consensus or after discussion with a third party.

### 2.6. Data Synthesis and Measures of Treatment Effect

The data were analyzed using Review Manager 5.3 software [28]. For outcomes, data regarding incidence were dichotomous, and others were continuous. Risk ratios (RRs) were calculated using the Mantel–Haenszel method for dichotomous outcomes, and weighted mean differences (MDs) were calculated using the inverse variance method for continuous variables. We used *I*^2^ statistics to assess the heterogeneity. A fixed-effect (FE) model was used if there was no significant heterogeneity in the data (*I*^2^ < 50%), and a random-effect (RE) model was used if significant heterogeneity was present (*I*^2^ > 50%) [30]. Clinical heterogeneity was assessed by reviewing the differences in the distribution of the participants' characteristics among trials, including age, gender, duration of disorder, and associated diseases. When heterogeneity was detected, we undertook sensitivity analyses to explore the influence of the risk of bias on effect estimates. The following aspects of quality will be considered for this sensitivity analysis: inadequate blinding and noncomparable groups because they had different baseline characteristics. Publication bias was assessed using funnel plots.

All the actual measures of the effect of continuous variables were the differences from baseline to endpoint.

## 3. Results

**Figure 1:**
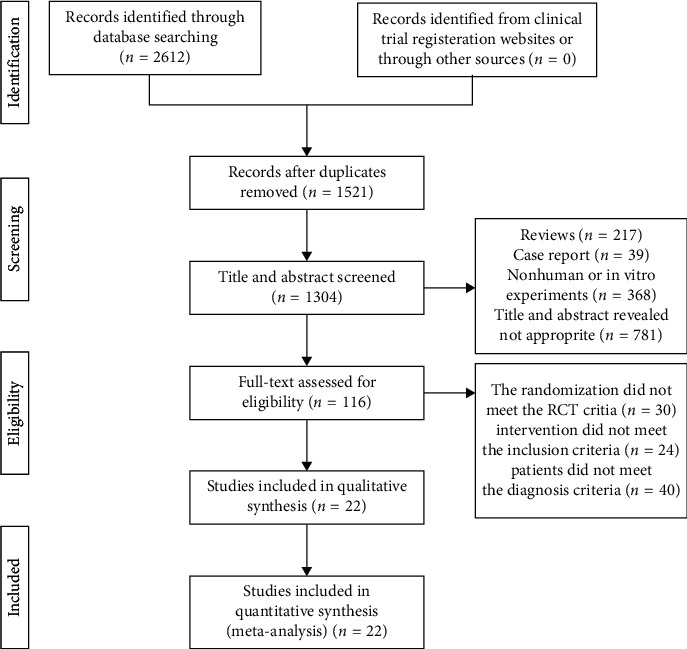
Flowchart of study selection phases of the systematic review of RCTs.

The flowchart of study search results is displayed in Figure 1. The primary searches identified a total of 2612 references using the search strategy. A total of 1521 articles were screened after 1091 duplicates of the same articles in different databases were removed. According to the inclusion criteria, 781 records were excluded based on the title and abstract because the title and abstract were not appropriate; 271 reviews were excluded; 39 case reports were excluded; and 368 nonhuman or in vitro experiment articles were excluded. After a detailed evaluation of the full text, additional 94 references were excluded. Finally, 22 RCTs [16, 18, 31–50] met the eligibility criteria and were included in the systematic review and meta-analysis.

### 3.1. Characteristics of the Included Trials

The characteristics of the included 22 trials are summarized in Table 1. A total of 3923 participants were involved, of which 1964 and 1959 were in the treatment and control groups. 10 trials were published in English, and 12 were published in Chinese. Except the one conducted in Iran and one conducted in Japan, all the remaining 20 trials were conducted in China. The trial sample size ranged from 34 to 514 participants. The mean age ranged from 39.6 to 60.7 years old, and gender was roughly balanced. This is consistent with the prevalence of the population, indicating that the 40–60 age group currently has the highest number of patients with IGT and diabetes (IDF 2019). The duration ranged from 8 weeks to 3 years, and the duration of 13 trials exceeded 6 months. All the trials adopted one or more conventional treatments, which were the same in both groups. Standard diagnostic diabetic criteria for prediabetes were applied to all included trials, including WHO 1999, WHO 2003, and ADA 2008. Furthermore, 2 trials used guidelines for the prevention and treatment of type 2 diabetes in China diagnostic criteria.

The trials reported random allocation of participants with prediabetes to the CHM intervention group versus control interventions (no treatment in 1 trial; placebo in 3 trials) or to CHM plus lifestyle management versus conventional medicine alone (lifestyle management in 13 trials; placebo plus lifestyle management in 3 trials; and conventional medicine plus LM in 2 trials). 12 of these studies mentioned syndromes of TCM, while in the remaining 10 studies, there were no descriptions of the relevant information. Three studies used the SF-36 health survey form to investigate the quality of life. Eight studies performed TCM symptomatic score analysis, while the other 15 did not mention the information. All the trials claimed a positive effect favoring CHM. The 22 randomized trials have been listed under characteristics of included studies (detailed in Table 1).

### 3.2. Adverse Events

Of the twenty-two included RCTs, 9 trials have reported the AEs, 11 trials clearly stated that no AEs occurred in either group, and 2 trials did not record the AEs. All reported AEs were mild-moderate; no serious AEs were reported. In Fang et al.'s study [18], AEs were noted in 14 patients, including 5 in the control group and 9 in the experimental group. 13 patients experienced gastrointestinal reactions, and one patient had pruritus. In Gao et al.'s study [16], 4 cases in the TCM intervention group showed mild abdominal distension; 3 cases in the control group showed mild abdominal distension. In Ke et al.'s study [33], 5 patients in the treated group felt fatigue, hunger, and dizziness, which were recovered after giving normal diets. In Shi et al.'s study [36], 1 patient in the JLD group experienced diarrhea, and 1 subject in the control group had nausea. No hypoglycemia or other serious AEs were reported. In An et al.'s study [42], there were 2 cases of nausea and anorexia, 1 case of mild diarrhea and 1 case of bowel ringing and stool change. In Ge et al.'s study [44], 8 patients had slight gastrointestinal discomfort within 1 week of medication, and the symptoms disappeared after symptomatic treatment, which did not affect the treatment. In Xing-yong and Mei-ling study [51], 1 patient in the CHM group withdrew due to nausea and bloating.

### 3.3. Risk of Bias in Included Studies

**Figure 2:**
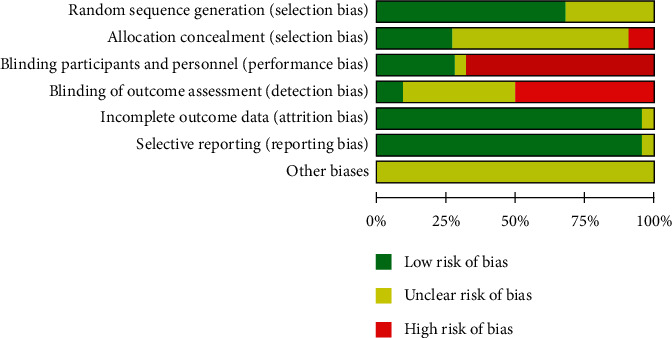
Risk of bias accessed using RevMan 5.3 according to the guidance in the Cochrane Handbook. Green represents low risk of bias, yellow represents unclear risk of bias, and red represents high risk of bias.

We used RevMan 5.3 to assess the risk of bias in included 22 studies. Of these 22 RCTs, 15 clearly specified the randomization procedure (random digital tables were used), and 4 of them described how the random number table is generated (software). Six studies used multicenter randomization; 1 multicenter study was conducted at Japan, while the rest 5 were conducted at China. Three studies described allocation concealment. There are 6 trials that used placebo to achieve satisfactory blinding effect, such as Karimi-Nazari et al. [32] and Lian et al. [34]. The rest 16 trials provided lifestyle management treatment or no treatment or conventional Western medicine treatment as control intervention, which makes these trials under high risk of bias. Only 2 studies reported the sample size estimation. 16 trials provided the number of withdrawals and dropouts in total. Other biases were difficult to assess in the selected RCTs as we were not able to get access to the trial protocols. The quality of all included studies is shown in Figure 2.

### 3.4. Incidence of Diabetes and Incidence of Normalization of Prediabetes

**Figure 3:**
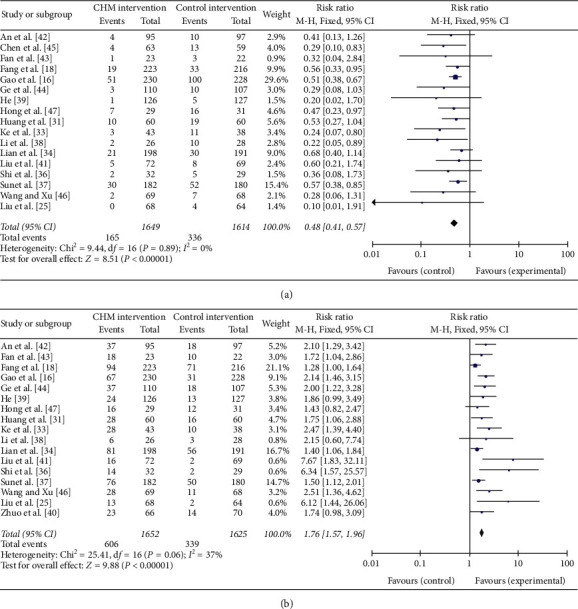
(a) Forest plot of the incidence of diabetes and (b) the incidence of normalization of prediabetes.

The incidence of diabetes refers to the number of participants who have developed type 2 diabetes according to the WHO or ADA standards at the end of the trial. 17 trials including 3263 patients (1649 in the CHM group while 1614 in the control group) reported the incidence of diabetes in each group (Figure 3). When the results of the 17 trials were aggregated, we found that compared to the control intervention, the CHM intervention can decrease the incidence of diabetes (RR = 0.48; 95% CI = (0.41, 0.57); test for overall effect: *Z* = 8.51; *P* < 0.001). The difference between the intervention group and the control group was statistically significant. In addition, the heterogeneity test showed that *I*^2^ = 0% and d*f* = 16 (*P* > 0.05), which suggest there was no heterogeneity in the incidence of diabetes among the 17 trials.

The incidence of normalization of prediabetes refers to the number of subjects returned to normal at the end of the test. 17 trials involving 3304 participants reported normalization of fasting blood glucose levels after intervention. In the 17 trials, the incidence of normalization of FBG among patients receiving CHM intervention was statistically higher than that of patients not receiving CHM intervention (RR = 1.76; 95% CI = (1.57, 1.96); test for overall effect: *Z* = 9.88, *P* < 0.001). The difference between the intervention group and the control group was statistically significant. In addition, the heterogeneity test showed that *I*^2^ = 37% and d*f* = 16 (*P* > 0.05), which suggest there was no heterogeneity in the incidence of normalization of FBG among the 17 trials.

### 3.5. FPG, 2hPG, and HbA1c

**Figure 4:**
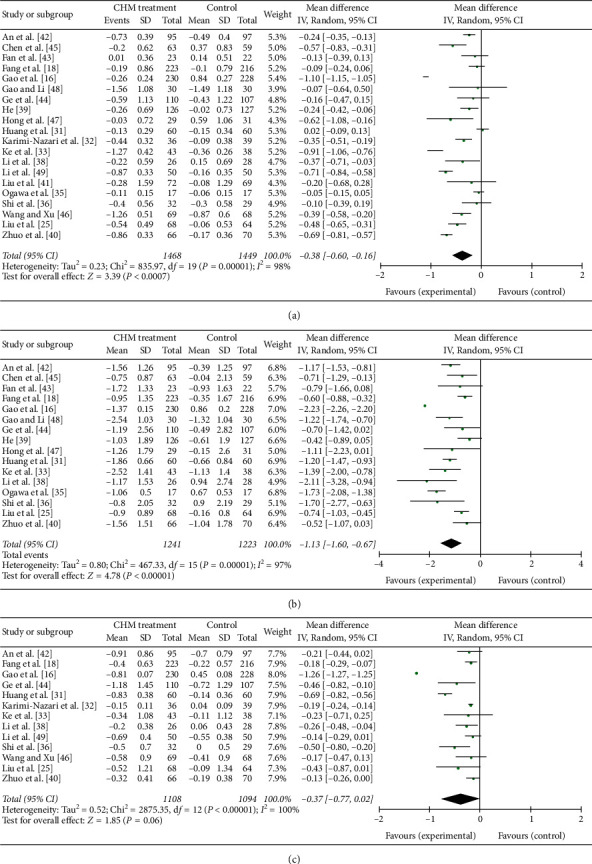
Forest plot of (a) FPG, (b) 2hPG, and (c) HbA1c.

20 trials including 2917 patients (1468 in the CHM group while 1449 in the control group) reported the FPG level in each group (Figure 4). The heterogeneity test showed that *I*^2^ = 98% and d*f* = 19 (*P* < 0.05), which suggest there was heterogeneity in the FPG level among the 22 trials, so a random-effect (RE) model was used. When the results of the 22 trials were aggregated, we found that compared to the control intervention, the CHM intervention can decrease the FPG level (MD = −0.38; 95% CI = (−0.60, −0.16); test for overall effect: *Z* = 3.39; *P* < 0.001). The difference between the intervention group and the control group was statistically significant.

16 trials including 2464 patients (1241 in the CHM group while 1223 in the control group) reported the 2hPG level in each group (Figure 4). The heterogeneity test showed that *I*^2^ = 97% and d*f* = 15 (*P* < 0.05), which suggest there was heterogeneity in the 2hPG level among the 17 trials, so a random-effect (RE) model was used. When the results of the 17 trials were aggregated, we found that compared to the control intervention, the CHM intervention can decrease the 2hPG level (MD = −1.13; 95% CI = (−1.60, −0.67); test for overall effect: *Z* = 4.78; *P* < 0.001). The difference between the intervention group and the control group was statistically significant.

13 trials including 2202 patients (1108 in the CHM group while 1094 in the control group) reported the HbA1c level in each group (Figure 4). The heterogeneity test showed that *I*^2^ = 100% and d*f* = 12 (*P* < 0.05), which suggest there was heterogeneity in the HbA1c level among the 14 trials, so a random-effect (RE) model was used. When the results of the 14 trials were aggregated, we found that there was no statistical difference between the CHM intervention and the control intervention group at the HbA1c level (MD = −0.37; 95% CI = (−0.77, 0.02); test for overall effect: *Z* = 1.85; *P* > 0.05).

When FPG, 2hPG, and HbA1c data were aggregated, considerable heterogeneity was found in these studies (*I*^2^ > 90%). This may be due to the type of intervention, duration of intervention, and different components of CHM.

### 3.6. TG, TC, and BMI

**Figure 5:**
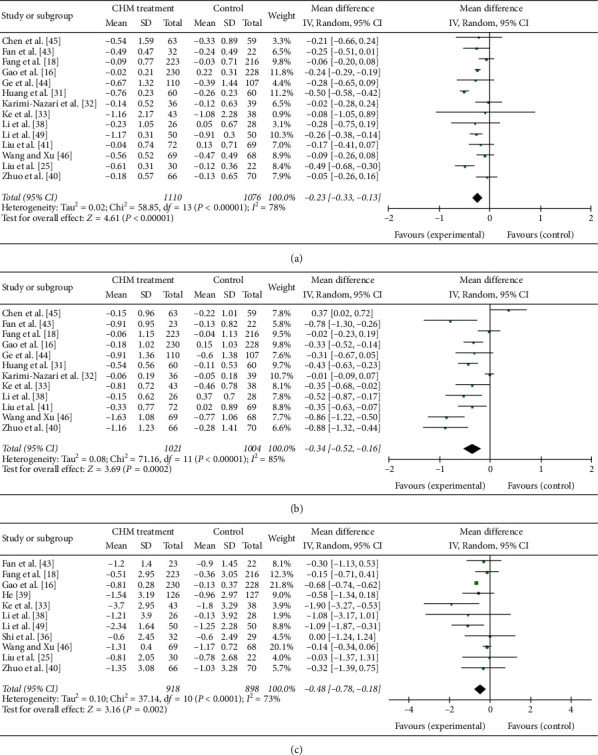
Forest plot of (a) TG, (b) TC, and (c) BMI.

14 trials including 2186 patients (1110 in the CHM group while 1076 in the control group) reported the TG level in each group (Figure 5). The heterogeneity test showed that *I*^2^ = 78% and d*f* = 13 (*P* < 0.05), which suggest there was heterogeneity in the TG level among the 14 trials, so a random-effect (RE) model was used. When the results of the 14 trials were aggregated, we found that compared to the control intervention, the CHM intervention can decrease the TG level (MD = −0.23; 95% CI = (−0.33, −0.13); test for overall effect: *Z* = 4.61; *P* < 0.001). The difference between the intervention group and the control group was statistically significant.

12 trials including 2025 patients (1021 in the CHM group while 1004 in the control group) reported the TC level in each group (Figure 5). The heterogeneity test showed that *I*^2^ = 85% and d*f* = 11 (*P* < 0.05), which suggest there was heterogeneity in the TC level among the 12 trials, so a random-effect (RE) model was used. When the results of the 12 trials were aggregated, we found that compared to the control intervention, the CHM intervention can decrease the TC level (MD = −0.34; 95% CI = (−0.52, −0.16); test for overall effect: *Z* = 3.69; *P* < 0.001). The difference between the intervention group and the control group was statistically significant.

11 trials including 1816 patients (918 in the CHM group while 898 in the control group) reported the BMI in each group (Figure 5). The heterogeneity test showed that *I*^2^ = 73% and d*f* = 10 (*P* < 0.05), which suggest there was heterogeneity in the BMI among the 11 trials, so a random-effect (RE) model was used. When the results of the 11 trials were aggregated, we found that compared to the control intervention, the CHM intervention can decrease the BMI (MD = −0.48; 95% CI = (−0.78, −0.18); test for overall effect: *Z* = 3.13; *P* < 0.01). The difference between the intervention group and the control group was statistically 

### 3.7. Publication Bias

**Figure 6:**
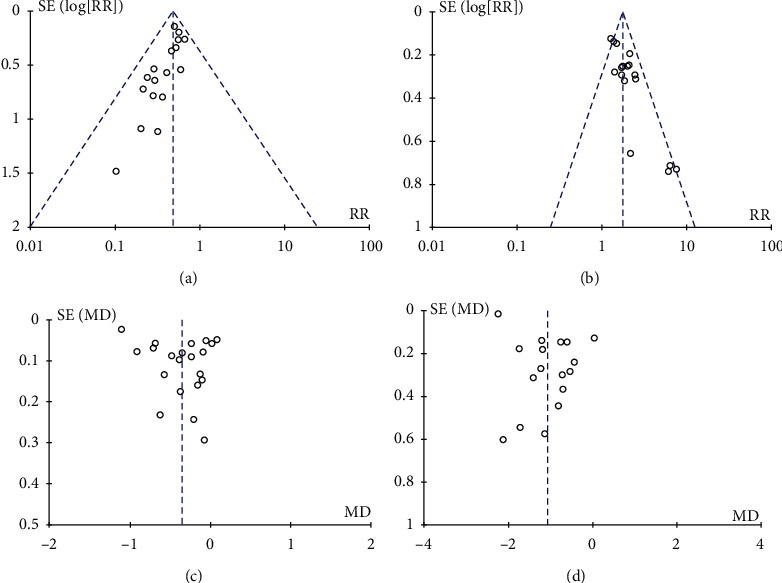
Funnel plot of publication bias. (a) Incidence of diabetes. (b) Incidence of normalization of prediabetes. (c) FPG. and (d) 2hPG.

Figure 6 shows the funnel plot of publication bias; the asymptomatic funnel plot of the incidence of diabetes and incidence of normalization of prediabetes suggests a publication bias; FPG and 2hPG are roughly symmetrical, suggesting that there is no obvious publication bias.

## 4. Discussion

As was known, the overall treatment philosophy of TCM is quite different from the Western medicine. Traditional Chinese medicine is holistic, and it pays attention to the balance between all aspects of the body [52]. Its principle is to treat the root cause of the disease, not the symptoms [53]. In this systematic review, we have evaluated the effectiveness and safety of CHM in the treatment of prediabetes among the selected 22 RCTs. A total of 3923 participants were involved, of which 1964 and 1959 were in the treatment and control groups. Overall, our findings upon the 22 RCTs showed that compared with the control group, CHM can decrease the incidence of diabetes (RR = 0.48; 95% CI = (0.41, 0.57); *P* < 0.001), increase the incidence of normalization of prediabetes (RR = 1.76; 95% CI = (1.57, 1.96); *P* < 0.001), and lower the level of FPG (MD = −0.38; 95% CI = (−0.60, −0.16]; *P* < 0.001), 2hPG (MD = −1.13; 95% CI = (−1.60, −0.67]; *P* < 0.001), TG (MD = −0.23; 95% CI = (−0.33, −0.13); *P* < 0.001), TC (MD = −0.34; 95% CI = (−0.52, −0.16); *P* < 0.001), and BMI (MD = −0.48; 95% CI = (−0.78, −0.18); *P* < 0.001), and there was no significant difference of HbA1c (*P* > 0.05).

In our study, there are two types of outcome data: dichotomous data (incidence of diabetes and incidence of FPG normalization) and continuous data (FPG, 2hPG, HbA1c, TG, TC, and BMI). In the results of the incidence of diabetes and incidence of FPG normalization, there is no significant statistical heterogeneity between the comparisons (incidence of diabetes: *I*^2^ = 0%; fasting blood glucose normalization: *I*^2^ = 37%). However, when FPG, 2hPG, HbA1c, TG, TC, and BMI data were aggregated, considerable heterogeneity was found in these studies (*I*^2^ > 70%). We have tried to do subgroup to solve this, but the heterogeneity still exists. This may be due to the type of intervention and duration of intervention, especially different components of CHM [54]. In the real clinical practice, it is important to note the clinical heterogeneity. CHM used in the 22 clinical trials has a wide range of components. These ingredients can be used for various clinical purposes, but they can still be considered as “classes” or “groups” of oral hypoglycemic herbs [55]. Nevertheless, our findings showed that compared with the control group, participants taking CHM are less likely to develop diabetes, more likely to have normal blood sugar, more likely to achieve lower FPG, 2hPg, TG, TC, and BMI after treatment, and have no difference between HbA1c.

As to the application of randomization methods, of these 22 RCTs, 15 clearly specified the randomization procedure (random digital tables were used), while the rest 7 articles did not provide any information about generating random assignment sequences. According to the previous surveys, articles that generated random assignment sequences accounted for 7.9% of all analyzed articles [56]. In the current meta-analysis, this proportion has increased a lot. We strongly recommend that researchers describe in detail the randomization method used in the article in future trials.

Distribution concealment is also an issue in clinical trials of traditional Chinese medicine. Previous studies reported that 0.3% of the articles described how the distribution concealment was achieved [56]. In this review, 3 of the 22 studies included described distribution concealment. Therefore, in future research, it is suggested to set up an independent data-monitoring committee to be responsible for the distribution and hiding process and to describe the implementation process of distribution and hiding in detail in the article. For Chinese researchers, establishing a national clinical practice center in China is an option for independent data monitoring.

Blind method is an important method to prevent the research results from being affected by placebo effect or observer bias [57]. The ideal blind method should cover participants, care providers, outcome assessors, and statistical analysts [58]. In this process, placebo plays an important role in blinding participants and nursing providers [59]. In our study, there are 6 trials adopted blinding methods by using placebo, such as placebo the Karimi-Nazari et al. [32] and Lian et al. [34] trials used placebo to achieve satisfactory blinding effect. The rest 16 trials provided lifestyle management treatment or no treatment or conventional Western medicine treatment as control intervention, which makes these trials under high risk of bias. One of the main reasons for not using placebos in these trials may be the difficulty in preparing the placebos with the same color and taste as the CHM used in the experiment group. A practical method is to prepare traditional Chinese medicine in the form of capsules or pills, making it difficult to distinguish placebo traditional Chinese medicine from real traditional Chinese medicine, such as Ogawa et al. [35] and Sun et al. [37] in our study. In short, researchers should pay attention to the quality of placebo in evaluating clinical trials of traditional Chinese medicine.

The chronic nature of prediabetes and diabetes indicates that people are more likely to receive long-term medical treatment, which could lead to the increase of adverse reactions [60]. CHM has a long history in treating diabetes and prediabetes in a wide and diverse population, so people had accumulated lots of experiences and knowledge about the safety of using CHM in treating diabetes [61]. However, all drugs have potential unexpected effects, including toxicity, so do herbs [62]. The adverse reactions of herbs may be intrinsic, such as predictable toxicity, overdose, and interaction with other herbs or idiopathic herbs (such as allergies), or they may also be external, related to misidentification, pollution, and lack of standardization [63]. So, in this systematic review, we did a detailed investigation of adverse reactions. In the twenty-two included RCTs, 9 trials have reported the AEs in total, and the AEs are mostly slight gastrointestinal discomfort. There were no obvious abnormalities in the safety indexes (hematuria routine, liver and kidney functions, ECG, etc.) in the intervention and control groups before and after treatment.

There were several limitations in our study. Although only RCTs were included in our study, the quality of the RCT design was not high. Of the 22 trials, 15 trials clearly specified the randomization procedure (random digital tables were used); only 3 studies described allocation concealment, and only 6 trials adopted blinding methods. It is difficult to draw a clear conclusion due to the differences among the ingredients of the Chinese medicine formulations included in the study. Traditional Chinese medicine emphasizes the syndrome differentiation, so the difference among diverse prescriptions and the difference in treatment are inevitable results of the essence of traditional Chinese medicine [64, 65]. This difference should be considered, which may be a factor leading to the heterogeneity in the continuous outcome measures [66]. Therefore, we should attach great importance to the quality of RCT research on CHM and encourage multicenter research to provide sufficient evidence and enhance its effectiveness. To achieve the consistency of the research conditions, we also encourage to use prescriptions including the same main ingredients to conduct the prediabetes research. These ingredients are included in an effective formula, such as ancient classic prescriptions and Chinese patent medicines. As far as the applicability of the evidence is concerned, of the 22 clinical trials included, 20 were conducted in China, and this feature will be restricted to a wider population. These methodological limitations with high risk of bias may be potential or inherent in clinical trials of traditional Chinese medicine.

## 5. Conclusion

In this study, there is preliminary evidence that CHM is beneficial in the treatment of prediabetes. Our findings upon the 22 RCTs showed CHM can statistically reduce the incidence of diabetes, increase the incidence of normalization of prediabetes, and lower the FPG, 2hPG, TG, TC, BMI levels, but with no significant difference in HbA1c. However, more rigorous RCTs are needed to confirm the findings and determine the safety, feasibility, and cost associated with CHM in the future. We encourage to conduct large-scale, multicenter, correctly randomized, placebo-controlled, and triple-masked trials to evaluate the efficacy of CHM in the treatment of prediabetes. The following factors should be emphasized in the future study: the sample size should be calculated before research; there should be a qualified randomization procedure; participants or outcome assessors should be assigned the blind methods, and detailed reports should be made; intention-to-treat (ITT) analysis [67] should be used to record dropouts or to analyze the results; and the report should follow the consolidated report test standard (CONSORT [68]).

## Figures and Tables

**Table 1:** Characteristics of the included 22 studies.

Study ID	Diagnosis criteria	Sample size	Age (mean ± SD)	Sex (male/female)	Loss	Experiment group	Control group	Duration	Outcome measures	Adverse effect
Fang et al. [18]	WHO 1999	Total: 514 I: 257/C: 257	I: 54.95 ± 9.50; C: 54.61 ± 10.51	I: 136/121 C: 142/115	I: 34/C: 41	CHM + LM	LM	12 months	①②③④⑤⑥⑦⑧⑨	AEs were noted in 14 patients, including 5 in the control group and 9 in the experimental group. 13 patients experienced gastrointestinal reactions, and one patient had pruritus. All reported AEs were mild-moderate; no serious AEs were reported.
Gao et al. [16]	WHO 1999	Total: 510 I: 255/C: 255	I: 49.3 ± 1.2; C: 51.12 ± 1.3	I: 110/145 C: 112/143	I: 25/C: 27	CHM + LM	LM	3 years	②③④⑤⑥⑦⑧⑨	4 cases in the TCM intervention group showed mild abdominal distension; 3 cases in the control group showed mild abdominal distension.
Huang et al. [31]	WHO 1999	Total: 127 I: 64/C: 63	I: 52.02 ± 8.60; C: 51.05 ± 9.25	I: 31/29 C: 35/25	I: 4/C: 3	CHM + LM	LM	12 weeks	①②③④⑤⑥⑦⑧⑨	No AEs occurred.
Karimi-Nazari et al. [32]	ADA	Total: 80 I: 40/C: 40	I: 57.95 ± 8.12 C: 57.9 ± 8.7	I: 13/23 C: 14/25	I: 4/C: 1	CHM	Placebo	8 weeks	③④⑥⑦⑧	No AEs occurred.
Ke et al. [33]	WHO 1999	Total: 85 I: 45/C: 40	I: 46.5 ± 7.3 C: 45.7 ± 7.5	I: 23/22 C: 20/20	I: 2/C: 2	CHM + LM	LM	6 months	②③④⑤⑥⑦⑧⑨	5 patients in the treated group felt fatigue, hunger, and dizziness, which were recovered after giving normal diets.
Lian et al. [34]	WHO 1999	Total: 420 I: 210/C: 210	I: 52.95 ± 10.06 C: 51.86 ± 10.16	I: 98/112 C: 106/104	I: 10/C: 18	CHM	Placebo	12 months	①②③	15 patients in the Tianqi group and 11 in the placebo group experienced AEs, all of which were mild adverse reactions (grades 1-2).
Ogawa et al. [35]	ADA	Total:34 I:17/C:17	I: 49.6 ± 1.5 C: 55.1 ± 2.0	I: 13/4 C: 13/4	I: 0/C: 0	CHM	Placebo	8 weeks	③④⑤	No AEs occurred.
Shi et al. [36]	WHO 2003	Total: 65 I: 34/C: 31	I: 47.1 ± 7.1 C: 49.9 ± 7.2	I: 17/17; C: 14/17	I:2/C:2	CHM + LM	LM	12 weeks	①②③④⑤⑥⑨	1 patient in the JLD group experienced diarrhea, and 1 subject in the control group had nausea. No hypoglycemia or other serious AEs were reported.
Sun et al. [37]	ADA 2008	Total: 362 I: 182/C: 180	I: 55.49 ± 8.61 C: 53.49 ± 8.85	I: 90/92 C: 82/98	I: 12/C: 16	CHM + LM	Placebo + LM	12 months	①②③	AEs in the treatment group and the control group occurred in 5 and 3 cases. No statistically significant difference was observed between the two groups.
Liu et al. [25]	WHO 1999	Total: 140 I: 70/C: 70	I: 51.3 ± 8.8 C: 50.7 ± 8.1	I: 31/39 C: 32/38	I: 6/C: 2	CHM	No treatment	6 months	①②③④⑤⑥⑦⑧⑨	No AEs occurred.
He [39]	WHO 1999	Total: 253 I: 126/C: 127	I: 29–70 C: 25–75	I: 64/62 C: 66/61	I: 0/C: 0	CHM + LM	LM	3 months	①②③④⑤⑨	No AEs occurred.
Liu et al. [41]	WHO 2008	Total: 150 I: 75/C: 75	No statistically significant difference	No statistically significant difference	I: 3/C: 6	CHM + LM	Placebo + LM	12 months	①②③④⑦⑧	No AEs occurred.
Zhuo et al. [40]	WHO 1999	Total: 136 I: 66/C: 70	I: 57.4 ± 10.2 C: 56.7 ± 10.9	I: 30/36 C: 34/36	I: 0/C: 0	CHM + LM	LM	12 weeks	②③④⑤⑥⑦⑧⑨	No AEs occurred.
An et al. [42]	ADA 2008	Total: 216 I: 108/C: 108	I: 41.8 ± 7.7 C: 39.6 ± 7.5	I: 70/38 C: 72/36	I: 13/C: 11	CHM + LM	LM	12 months	①②③④⑤⑥	In the CHM group, drug-related adverse reactions occurred in 5 cases, mostly mild, and the patients could be relieved by themselves. There were 2 cases of nausea and anorexia, 1 case of mild diarrhea, and 1 case of bowel ringing and stool change
Li et al. [38]	GPTDC	Total: 64 I: 31/C: 33	I: 55.7 ± 12.8 C: 56.93 ± 10.34	I: 5/21 C: 6/22	I: 5/C: 5	CHM + LM	LM	12 months	①②③④⑤⑥⑦⑧⑨	No AEs occurred.
Fan et al. [43]	WHO 1999	Total: 51 I: 25/C: 26	I: 54.60 ± 9.09; C: 57.45 ± 8.76	I: 10/13 C: 11/11	I: 2/C: 4	CHM + LM	LM	6 months	①②④⑤⑥⑦⑧⑨	No records.
Ge et al. [44]	ADA 2009	Total: 234 I: 117/C: 117	60.7 ± 8.1	141/93	I: 10/C: 17	CHM + LM	LM	12 months	①②③④⑤⑥⑦⑧	8 patients had slight gastrointestinal discomfort within 1 week of medication, and the symptoms disappeared after symptomatic treatment, which did not affect the treatment
Chen et al. [45]	WHO 1999	Total: 122 I: 63/C: 59	I: 52.87 ± 10. 57 C:52.97 ± 10. 94	I: 27/36 C: 32/27	I: /C:	CHM + LM	placebo + LM	12 months	①③④⑤⑦⑧	No AEs occurred.
Wang and Xu [46]	GPTDC	Total: 140 I: 70/C: 70	I: 43.7 ± 5.8 C: 42.9 ± 6.1	I: 27/36 C: 32/27	I: 1/C: 2	CHM + LM	Metformin + LM	3 months	①②③④⑥⑦⑧⑨⑩	1 patient in the CHM group withdrew due to nausea and bloating.
Hong et al. [47]	WHO 1999	Total: 60 I: 29/C: 31	I: 44 ± 9.3 C: 43 ± 10.1	I: 10/19 C: 10/21	I: 0/C: 0	CHM + LM	LM	3 months	①②③④⑤⑩	No AEs occurred.
Gao and Li [48]	WHO 1999	Total: 60 I: 30/C: 30	I: 49.0 ± 5.3 C: 50.2 ± 6.3	I: 20/10 C: 18/12	I: 0/C: 0	CHM + LM	LM	6 months	④⑤	No records.
Li et al. [49]	ADA 2010	Total: 100 I: 50/C: 50	I: 52.70 ± 7.28 C:50.80 ± 7.05	I: 24/26 C: 13/27	I: 0/C: 0	CHM + LM	Metformin + LM	3 months	③④⑥⑦⑨	No AEs occurred.

① Incidence of type 2 diabetes mellitus; ② incidence of the normalization of blood glucose; ③ AEs; ④ FPG; ⑤ 2hPG; ⑥ HbA1c; ⑦ TG; ⑧ TC; ⑨ BMI.

## Data Availability

The data used to support the findings of this study are available from the corresponding author upon request.
